# Choline Metabolism Alteration: A Focus on Ovarian Cancer

**DOI:** 10.3389/fonc.2016.00153

**Published:** 2016-06-22

**Authors:** Marina Bagnoli, Anna Granata, Roberta Nicoletti, Balaji Krishnamachary, Zaver M. Bhujwalla, Rossella Canese, Franca Podo, Silvana Canevari, Egidio Iorio, Delia Mezzanzanica

**Affiliations:** ^1^Unit of Molecular Therapies, Department of Experimental Oncology and Molecular Medicine, Fondazione IRCCS Istituto Nazionale dei Tumori, Milan, Italy; ^2^Russell H. Morgan Department of Radiology and Radiological Science, Division of Cancer Imaging Research, In Vivo Cellular and Molecular Imaging Center, The Johns Hopkins University School of Medicine, Baltimore, MD, USA; ^3^Department of Cell Biology and Neurosciences, Istituto Superiore di Sanità, Rome, Italy; ^4^Functional Genomics and Informatics, Department of Experimental Oncology and Molecular Medicine, Fondazione IRCCS Istituto Nazionale dei Tumori, Milan, Italy

**Keywords:** choline kinase, ovarian cancer, phosphocholine metabolism, reversal of drug resistance, antioxidant defense

## Abstract

Compared with normal differentiated cells, cancer cells require a metabolic reprograming to support their high proliferation rates and survival. Aberrant choline metabolism is a fairly new metabolic hallmark reflecting the complex reciprocal interactions between oncogenic signaling and cellular metabolism. Alterations of the involved metabolic network may be sustained by changes in activity of several choline transporters as well as of enzymes such as choline kinase-alpha (ChoK-α) and phosphatidylcholine-specific phospholipases C and D. Of note, the net outcome of these enzymatic alterations is an increase of phosphocholine and total choline-containing compounds, a “cholinic phenotype” that can be monitored in cancer by magnetic resonance spectroscopy. This review will highlight the molecular basis for targeting this pathway in epithelial ovarian cancer (EOC), a highly heterogeneous and lethal malignancy characterized by late diagnosis, frequent relapse, and development of chemoresistance. Modulation of ChoK-α expression impairs only EOC but not normal ovarian cells, thus supporting the hypothesis that “cholinic phenotype” is a peculiar feature of transformed cells and indicating ChoK-α targeting as a novel approach to improve efficacy of standard EOC chemotherapeutic treatments.

## Introduction

The uncontrolled cell growth characteristic of neoplastic diseases, besides involving deregulated control of cell proliferation, requires an adjustment of energy metabolism to sustain cell growth and division. Altered energy metabolism is considered as widespread in cancer cells as other cancer-associated characteristics. Reprograming of cell metabolism has been therefore included among cancer hallmarks (1), a series of biological properties acquired by tumor cells during transformation and disease progression. Metabolites themselves can interfere with oncogenic-driven cell signaling (2) and, since cancer cells are dependent on these changes in metabolism, these altered pathways represent attractive sources of promising therapeutic targets (3, 4). Furthermore, the differential uptake in some human cancers of glucose, choline, acetate, methionine, and aminoacid analogs, when used as radiotracers in positron emission tomography (PET) imaging, is considered a clinically useful diagnostic/staging tool (4).

## Choline Metabolism Alteration in Human Cancer

**Figure 1:**
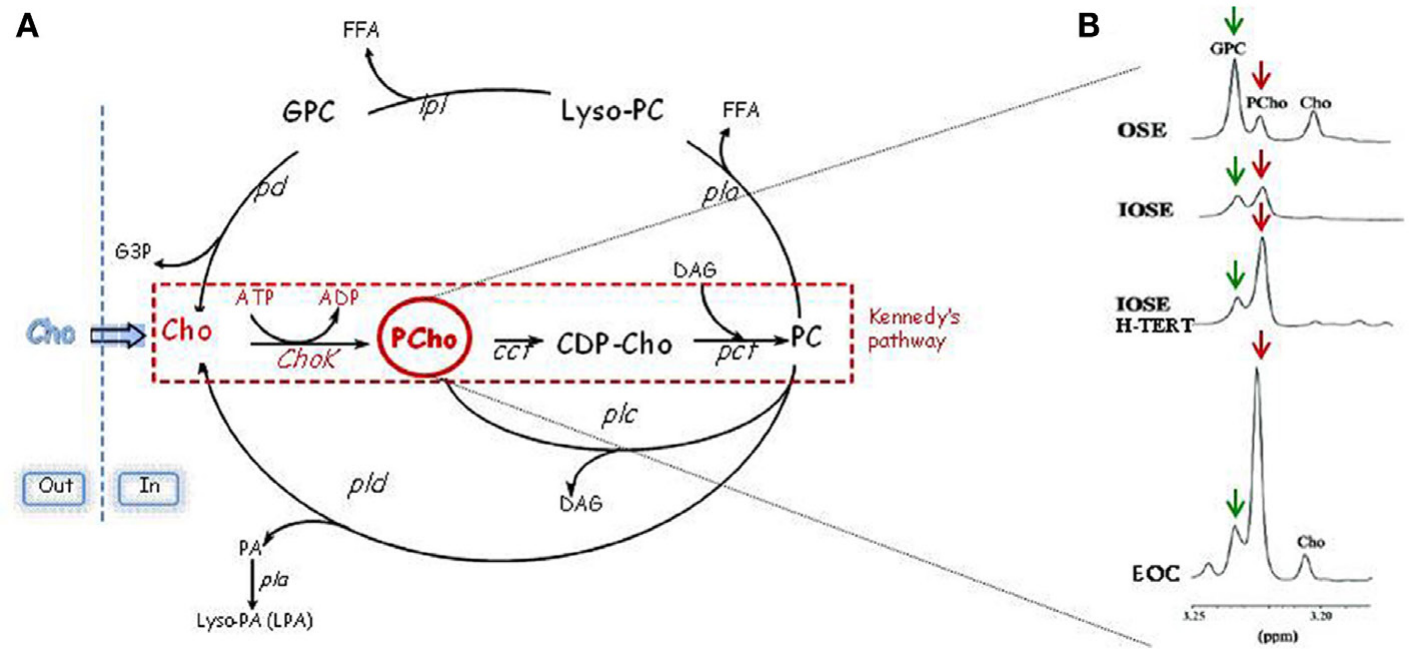
**Schematic representation of PC cycle and routes of PCho accumulation**. **(A)** Kennedy’s pathway: Cho, choline; Chok, choline kinase; PCho, phosphocholine; CCT, cytidylyltransferase; CDP-Cho, cytidine diphosphate-choline; PCT, phosphocholine phosphotransferase; DAG, diacylglycerol; PC: phosphatidylcholine. Catabolic pathways: PLC, PC-specific phospholipase C; PLD, PC-specific phospholipase D; PLA, PC-specific phospholipases A2 and A1; PD, phosphodiesterase; GPC, glycerophosphocholine; PA, phosphatidate. **(B)** In the progression from non-tumoral ovarian surface epithelial cells (OSE) or immortalized cell variants (IOSE with an increased replicative potential and IOSE-HTERT with unlimited replicative potential) to EOC cells, there is an evident accumulation of PCho (red arrows) and a decrease of GPC (green arrows).

Aberrant choline metabolism, characterized by increased phosphocholine (PCho) and total choline-containing compounds (tChos), is a fairly new metabolic hallmark that can be monitored in cancer by magnetic resonance spectroscopy (MRS) and that reflects the complex reciprocal interactions between oncogenic signaling and cellular metabolism (5, 6). PCho is both a precursor and a breakdown product of phosphatidylcholine (PC), one of the major components of cellular membranes. Indeed, the PCho content is sustained by activation of enzymes involved in PC biosynthetic and catabolic pathways: choline kinase (ChoK) and PC-specific phospholipase C (PC-PLC) (Figure 1A). ChoK is the first enzyme of the Kennedy pathway responsible for catalyzing the phosphorylation of free choline to form PCho in the biosynthesis of PC (7). Three isoforms of ChoK are present in mammalian cells encoded by two different genes: choline kinase-alpha (*CHKA*) and choline kinase-beta (*CHKB*). However, only ChoK-alpha (ChoK-α) has a central role in sustaining PC biosynthesis required for the uncontrolled growth of cancer cells, and ChoK-β alone cannot compensate this activity (8). In addition to its metabolic function, ChoK-α has been proven to play a critical role in oncogenesis, tumor progression, and metastasis of several cancers being required for the activation of growth factor-triggered signaling pathways (Ras activation, PI3K signaling), roles that proposed CHKA as an oncogene (5, 9–11). Indeed, an altered choline metabolism, sustained by increased expression and activity of ChoK-α, has been reported in various human malignancies (12–19). Furthermore, in the case of early stage non-small cell lung cancer (20), early stage hepatocellular carcinoma (21), and prostate cancer (22), a prognostic role of ChoK-α overexpression has been revealed. These observations provided the molecular basis for the development of non-invasive imaging approaches based on choline phosphorylation for the characterization of tumor growth and response to therapy (23–26) as well as the rationale for developing specific inhibitors for this metabolic pathway even in diseases other than cancer (27, 28).

## Choline Kinase as a Potentially New Therapeutic Target for Cancer Treatment

Choline kinase-α is an enzyme of particular interest being at the crossroad of the main survival signaling pathways with its overexpression contributing, through a positive feedback loop, to increased MAPK and PI3K signaling (5, 29). A large body of work in cancer cells suggests that ChoK-α expression and activity is directly associated not only with increased cancer cell proliferation but also with malignancy, making it a potential novel target for image-guided cancer therapy. In fact, the targeting of ChoK-α by RNA interference (RNAi) results in decreased PCho and tCho levels in human breast cancer cells while leaving human mammary epithelial cells unaffected (30, 31), thus opening an important therapeutic window for the development of a pharmacological intervention directed to this enzyme (32, 33). Indeed, the antitumor effects of ChoK-α inhibition has been reported in various cancers (34–40). Different compounds are at the moment available for ChoK-α pharmacological inhibition: hemicholinium-3 (HC-3), a competitive inhibitor with a ChoK-α mimetic structure able to block also choline transport, is very efficient *in vitro* but highly toxic *in vivo* (41); MN-58b, not commercially available, is a less toxic HC-3 derivative able to inhibit cell growth *in vivo* in animal models (42); RSM-932A, a Chok-α inhibitor selected for further clinical development due to its potent *in vivo* anticancer activity and lack of toxicity at the effective doses (43); CK37, a small molecule able to inhibit tumor growth in preclinical models (44); and new small molecule inhibitors identified by fragment-based drug discovery (45). These data suggest that inhibiting ChoK-α, even in combination with standard chemotherapeutic treatments, might represent a new anticancer approach particularly in tumors such as ovarian cancer with a still open clinical need for the identification of more efficient therapeutic modalities.

## Ovarian Cancer

Epithelial ovarian cancer (EOC) is a life-threatening disease characterized by late-stage presentation and a distinctive ability to heavily invade the abdominal cavity (46). The yearly worldwide incidence of this cancer is of 238,700 new cases with a global mortality of 151,900 deaths per year (47), which make EOC the leading cause of death for gynecological cancers. Standard treatment for EOC patients is an aggressive primary surgery followed by platinum-based chemotherapy. However, around 30% of the patients undergo chemotherapeutic treatments before being identified as chemoresistant, and even for patients who achieve a pathological complete response, maintaining disease-free status remains a challenge. Indeed, most of the patients develop platinum-resistant recurrent disease, a largely incurable state. Despite the impressive improvement of surgical approaches and drug development, survival rate has changed little in the last decades (48), and 5-year survival rate for advanced stage patients is still around 30% (49).

It is well known that resistance to chemotherapy is one of the tumor “hallmarks” that also includes tumor ability to modify/reprogram cellular metabolism (1) to face with the biosynthetic demand of rapid proliferation and to overcome metabolic stress imposed by the microenvironment. As for many other cancer types, also EOC cells become dependent on these metabolic changes, which could be possibly exploited to identify therapeutic targets for overcoming chemoresistance.

## Targeting the Altered Choline Metabolism to Evade/Circumvent EOC Chemoresistance

Aberrant choline metabolism has been recently defined also in EOC. Analysis of expanded tCho MR spectral profiles showed that the relative areas of signal components due to individual choline metabolites [glycerophosphocoline (GPC), PCho, and free choline] changed in the progression from non-tumoral ovarian surface epithelial cells (OSE) or immortalized cell variants to EOC cells (15), with the PCho relative signal becoming predominant in carcinoma cells (Figure 1B).

A large body of work demonstrated that, in EOC, these alterations are sustained by the activation of two enzymes ChoK-α and PC-PLC, respectively, involved in the PC biosynthetic and catabolic pathway. ChoK-α has a major role in increasing PCho content. Indeed, ChoK-α is overexpressed and hyperactivated in EOC cells as compared with the normal counterpart, accounting for up to the 70–80% of the total intracellular PCho content (16, 37). Gene expression analysis of the enzymes involved in the PC anabolic pathway showed that only CHKA was overexpressed, whereas the expression of other enzymes involved in the Kennedy pathway, as well as the beta isoform of choline kinase (CHKB), choline transporters, and enzymes involved in some catabolic pathways (mediated by PLD, PLA1, and PLA2) remained essentially unchanged (16). Among the enzymes involved in catabolic pathways, only PC-PLC is directly involved in PCho production. Although the mammalian PC-PLC has not been currently cloned and its sequence is unknown, this enzyme has been shown to be overexpressed and hyperactivated in EOC cells compared with normal counterparts (16, 50). EOC cells exposure to the PC-PLC inhibitor D609 abolished the activity of this enzyme and reduced the intracellular PCho level (without altering GPC and free choline contents), suggesting that also PC-PLC partially contributes to the intracellular PCho pool in EOC (16, 50).

**Figure 2:**
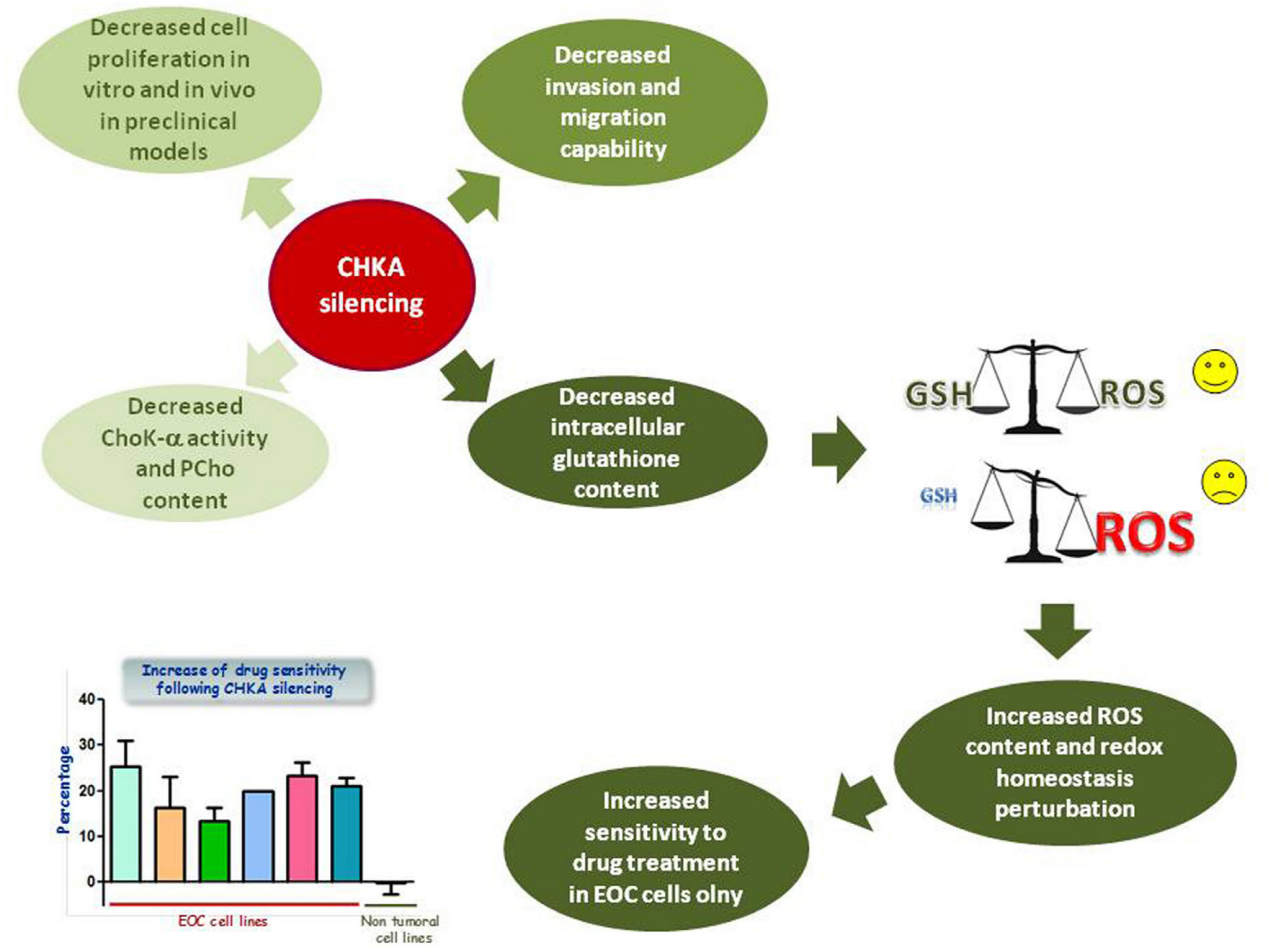
**Effects of CHKA silencing in EOC cell lines**. CHKA silencing decreases cell proliferation *in vitro* and *in vivo* in preclinical models, impairs the capability of EOC cells to migrate and invade, and decreases levels of intracellular GSH, thus impairing cellular redox homeostasis and therefore increasing EOC, but not IOSE-HTERT sensitivity to platinum treatment.

To define the role of the abnormal expression and increased activation of ChoK-α in EOC biology, the enzyme was inactivated by transient and stable RNA interference in EOC cell lines and in non-tumoral immortalized cell variants. ChoK-α inhibition resulted in a less aggressive phenotype (36, 37), causing a decreased cell proliferation both *in vitro* and in preclinical *in vivo* models of Nu/Nu mice, an impaired capability to migrate and invade, together with an increased sensitivity to drug treatment of EOC cells (Figure 2).

The effects related to CHKA targeting in EOC appeared to induce a perturbation on EOC cell behavior different than that observed in other cellular models. In fact, neither previously described reduction of Akt phosphorylation in a PI3K-independent way (51) nor an attenuation of MAPK and PI3K/AKT signaling (29) was observed. Furthermore, in spite of a reduced cell proliferation, neither a decrease of cell viability nor apoptosis was detected in *CHKA*-silenced EOC cells.

On the other hand, the analysis of global metabolic profiling identified an altered glutathione (GSH) metabolism characterized by a decreased cysteine and GSH content (37). GSH is a thiol peptide involved in regulation of cell redox status through its antioxidant activities (52). Reduction of GSH content, perturbing redox homeostasis, is expected to render tumor cells more susceptible to chemotherapeutic treatment (Figure 2), and high intracellular levels of reduced GSH have been shown to contribute in developing resistance to chemotherapeutic drugs including platinum and doxorubicin (53–55). Accordingly, CHKA targeting in different EOC cellular models increased reactive oxygen species (ROS) intracellular levels and sensitivity to platinum and doxorubicin treatment. These effects were mediated by the reduction of GSH content, even in a drug resistant EOC model, while leaving unaffected the non-tumoral immortalized epithelial ovarian cells (37) (Figure 2).

Interestingly, the critical enzyme cleaving GPC to produce choline, the initial step in the pathway controlling the GPC/PC ratio, has been recently identified (56). The enzyme, named endometrial differential 3 (EDI3), was initially described in a breast cancer cell line, where its inhibition corrected the GPC/PC ratio and reduced migratory activity of tumor cells. Also, EDI3 overexpression was associated with higher risk of developing metastasis and decreased survival in endometrial and ovarian cancer (56). The recent finding that EDI3 links choline metabolism to integrin expression, cell adhesion, and spreading also in an EOC cell line (57), suggests EDI3 as a new possible target to be explored, further confirming the value of the choline metabolism for therapeutic intervention in EOC.

The EOC “*cholinic phenotype*” is a peculiar feature of transformed cells that recapitulates the addiction of EOC cells to GSH content for the maintenance of their antioxidant defense. Targeting mechanisms upon which cancer cells are expected to be dependent (CHKA expression and cellular ROS homeostasis) could explain the differential response of cancer and non-transformed cells to CHKA knockdown. As well known, cancer cells acquire specific genetic and epigenetic alterations that involve hyperactivation of oncogenes and/or inactivation of oncosuppressor genes. Some genetic changes support survival of cancer cells by creating specific signaling, which sustain metabolic pathways. However, the overall deregulation of cellular processes and functions is frequently associated with enhanced cellular stress, and malignant cells have to adapt to this phenotype, becoming dependent on a number of non-oncogenic functions to survive (3). Similarly, a dependency associated with ROS homeostasis has been shown to constitute a selective liability of malignant cells also in xenograft tumor models (58). Identifying such dependencies represent a promising alternative for the development of new therapeutic strategies to successfully target metabolic enzymes minimizing adverse effects on normal tissues. Synergisms of choline metabolism knockdown with conventional treatment might open an interesting clinical perspective, as it could represent an alternative strategy to increase the treatment efficacy also by reducing the clinical dose of drugs and limiting the damage of normal cells.

## Exploiting the Altered Choline Metabolism for the *In Vivo* Imaging of Tumor Response to Therapies

Due to the increased metabolic activity, tumors are expected to intake greater amounts of a radioactive tracer than the adjacent normal tissues, justifying the use of PET imaging to monitor response to treatment and disease recurrence (59). Accordingly, the increased expression and activity of ChoK-α and choline transporters in tumor cells promoted a rapid development of radiolabeled choline analogs, as PET imaging tracers and ^11^C- or ^18^F-choline were proven to be more effective than ^18^F-fluorodeoxyglucose (FDG), whose abundant radioactivity excretion into the bladder could hamper image interpretation. Indeed, in the case of radiolabeled choline analogs, their incorporation mainly reflects the total amount of radiotracer that enters the cell by choline transport and accumulates, by efficient phosphorylation mainly due to ChoK activation, in the pool of water-soluble intermediates of the Kennedy pathway (24). Within the time window of choline PET examinations, the contributions given by PC catabolic enzymes (such as phospholipases C and D) to the pool of these radiolabeled choline derivatives are instead negligible (6).

The large proportion of studies evaluating choline radiotracers has been conducted in prostate cancer where choline PET gave a clinical contribution in the diagnosis and monitoring of response to therapy; however, the utility of ^11^C- or ^18^F-choline as radiotracers has been extensively reported also in non-prostate histotypes (24, 59). Although studies on ^11^C- or ^18^F-choline as radiotracers in PET examinations of genitourinary tract cancers are currently under active evaluation as an alternative to ^18^F-FDG, few studies are currently available on the use of choline-based tracer in ovarian cancer, even at preclinical level (60, 61).

In spite of the EOC cholinic phenotype, a choline-based non-invasive detection and management is still a relatively unexplored field in this disease. An improvement in instrumentation and the integration of different imaging approaches, such as PET, magnetic resonance imaging (MRI), and computed tomography (CT), could provide the unique opportunity to monitor both morphologic and metabolic changes in tumor to improve diagnosis and the assessment of therapeutic efficacy.

## Author Contributions

All the authors have contributed in writing this mini review and have been directly involved in obtaining the results described in the chapter dedicated to ovarian cancer and choline metabolism. All the authors reviewed the manuscript and approved the final version.

## Conflict of Interest Statement

The authors declare that the research was conducted in the absence of any commercial or financial relationships that could be construed as a potential conflict of interest.
